# Practical Strategies to Reduce Ochratoxin A in Foods

**DOI:** 10.3390/toxins16010058

**Published:** 2024-01-20

**Authors:** Hyun Jung Lee, Hae Dun Kim, Dojin Ryu

**Affiliations:** 1 Department of Animal, Veterinary and Food Sciences, University of Idaho, Moscow, ID 83844, USA; haedun91@gamil.com; 2 Division of Food, Nutrition and Exercise Sciences, University of Missouri, Columbia, MO 65211, USA; dryu@missouri.edu

**Keywords:** ochratoxin A (OTA), toxicity, reduction, food processing, food additives

## Abstract

Ochratoxin A (OTA), a potent nephrotoxin, is one of the most deleterious mycotoxins, with its prevalence in agricultural crops and their processed foods around the world. OTA is a major concern to food safety, as OTA exposure through dietary intake may lead to a significant level of accumulation in the body as a result of its long half-life (about 35 days). Its potent renal toxicity and high risk of exposure as well as the difficulty in controlling environmental factors OTA production has prompted the need for timely information on practical strategies for the food industry to effectively manage OTA contamination during food processing. The effects of various food processes, including both nonthermal and thermal methods, on the reduction in OTA were summarized in this review, with emphasis on the toxicity of residual OTA as well as its known and unknown degradation products. Since complete removal of OTA from foodstuffs is not feasible, additional strategies that may facilitate the reduction in OTA in food, such as adding baking soda and sugars, was also discussed, so that the industry may understand and apply practical measures to ensure the safety of its products destined for human consumption.

## 1. Introduction

**Figure 1:**
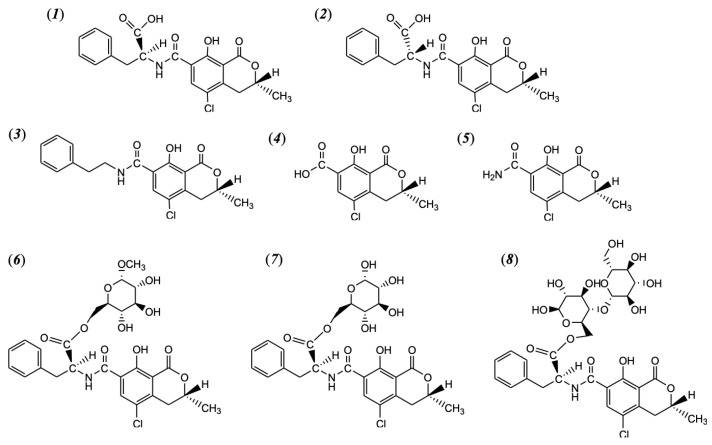
Chemical structure of 2S′-ochratoxin A (2S′-OTA, generally called OTA, called 14-(S)-ochratoxin A in the past) (1); 2R′-ochratoxin A (2R′-OTA, called 14-(*R*)-ochratoxin A in the past, as known as OTA isomer) (2); 14-decarboxyl-ochratoxin A (DC-OTA) (3); ochratoxin α (4) and ochratoxin α amide (5); ochratoxin A-methyl-a-D glucopyranoside ester (6); ochratoxin A glucose ester (7); and ochratoxin A cellobiose ester (8).

Ochratoxin A (OTA, Figure 1) has received considerable attention as one of the most frequently occurring mycotoxins in the world along with aflatoxins, fumonisins, deoxynivalenol, and zearalenone. OTA is a potent renal carcinogen based on experimental animal studies and thus is classified as a Group 2B possible human carcinogen [1,2,3,4]. OTA is also known to be a mutagenic, teratogenic, hepatotoxic, and immunosuppressive mycotoxin [5,6].

One of the most significant contributing factors in its worldwide occurrence is the diversity of OTA producers—i.e., numerous species in two ubiquitous yet distinctively different two fungal genera of *Aspergillus* and *Penicillium*, that are collectively capable of growing and producing OTA at a wide range of temperatures (0–37 °C). Therefore, these fungi and OTA have been found in all major agricultural commodities worldwide, and, due to its heat stability, OTA has been found in an exceptionally wide variety of processed foods including all major cereal grains, beans, coffee, wine, beer, fresh and dried fruits (e.g., grapes, sultanas, raisins, and dates), fruit juices, cocoa, nuts, spices, meats (pork and poultry), milk, infant formula and infant cereals [7,8,9,10,11,12,13,14,15,16,17]. In addition, the extremely long half-life of OTA in humans (ca. 35 days) is of significant concern, in that the exposure could exceed safe levels while OTA may be accumulated, remain in the organs and blood, and even be transmitted to milk [18,19].

In common with other major mycotoxins, foods with no OTA may be obtained only by protecting crops from the contamination of toxigenic fungi in the field and during storage [20]. However, this is very difficult as the OTA-producing fungi are ubiquitous in nature worldwide and their contamination and subsequent toxin production may not be prevented. It should be noted that the presence of the toxigenic fungi may not correlate with the level of contamination as the environmental conditions could limit the production of OTA. It is also possible to detect OTA in raw agricultural materials without the presence of or actively growing OTA producers, since chemical processes or environmental changes in the field and storage can inactivate the fungi but not alter OTA that remains in the commodity [21,22]. Once OTA enters the food supply chain, unfortunately, the most attainable strategy to reduce the deleterious effects of OTA is reducing the concentration of toxin during food processing, i.e., before it reaches consumers.

There are two main ways for reducing OTA in foods for human consumption that the food industry can consider: non-thermal and thermal food processing. Regardless of the route, the method of choice should be technically feasible, scalable, and economical. In addition, it must not generate toxic by-products, known and/or unknown, while not causing issues with other food quality parameters such as flavor, taste, color, and nutritional content. Therefore, practical processing technologies that the food industry can adopt to reduce OTA and additional strategies to mitigate its toxicity are discussed in this article.

## 2. Ochratoxin A (OTA)

### 2.1. Chemistry

OTA (*N*-[(3*R*)-(5-chloro-8-hydroxy-3-methyl-1-oxo-7-isochromanyl) carbonyl]-L-phenylalanine, C_20_H_18_ClNO_6_, MW = 403.8, CAS No. 303-47-9) is a chlorinated isocoumarin with a pentaketide skeleton that links to L-phenylalanine via an amide bond (Figure 1). OTA is a white, crystalline compound, highly soluble in polar organic solvents and slightly soluble in water but insoluble in petroleum esters [5]. OTA is weakly acidic, with pKa values in the ranges of 4.2–4.4 and 7.0–7.3 for the carboxyl group and the phenolic hydroxyl group, respectively [23,24,25]. OTA is fairly stable in foods during thermal processes with its melting point of 169 °C [26,27]. Only a few analogs or degradation products have been identified to date, as shown in Figure 1. These known compounds include 14-decarboxyl OTA and 14-(R)-OTA, detected in commercial roasted coffee beans and coffee drinkers’ blood’s in trace amounts [28]. According to several reports [29,30,31,32], matrix bounded forms such as esters, as well as OTα and OTα amide, were formed when coffee beans were roasted at 240 °C for 9 min. It should be noted that these compounds were detected in roasted coffee, i.e., no other products or processes were subjected to investigate the reaction kinetics and mechanisms.

### 2.2. Toxicology and Toxicity Mechanism

The major target organ of OTA is the kidney and OTA is classified as a possible human carcinogen, Group 2B [1,2,3,4]. OTA is also known to be a mutagenic, teratogenic, hepatotoxic, and immunosuppressive mycotoxin [5,6]. Due to its limited solubility in water and affinity to proteins, OTA binds tightly to blood plasma proteins. Hence, OTA may be reabsorbed in the kidney and recirculated instead of excreted like other xenobiotics, resulting in delayed biotransformation or renal clearance, with a prolonged half-life of about 35 days in humans, the longest of the known xenobiotics for mammals [19,33].

The chemical carcinogenesis or exact mechanism of OTA toxicity has not been understood clearly yet. There are several proposed mechanisms related to OTA toxicity: (a) inhibiting mitochondrial respiration and ATP production [34,35]; (b) the inhibition of protein synthesis [36,37,38,39]; (c) DNA-strand breakages [40,41]; (d) lipid peroxidation [42,43,44]; and (e) oxidative stress [40,41,45,46,47,48]. Based on the toxicological data available to date, oxidative stress appears to be the most plausible underlying mechanism of toxicity of OTA either directly or indirectly [49,50], which would lead to a broader systemic as well as organ-specific toxicities.

The toxicity of OTA has been linked to free radical-mediated oxidative cell damage or oxidative stress. Schaaf et al. [48] suggested that OTA-induced reactive oxygen species (ROS) cause proximal tubule cell damage that can lead to a wide range of lesions in cell components. OTA exposure increased not only ROS levels but also oxidative enzymes for detoxification through PXR and AhR pathways [51,52]. OTA is also known to not only reduce the expression of multiple genes related with antioxidant defenses in the body, including Nrf2, which is involved in both the basal expression, but in the induction of genes for detoxification and antioxidant enzymes [46,53,54,55]. It is likely that the reduced expression of these genes results in decreased antioxidant defense and in turn increased oxidative stress and macromolecular damage. In contrast, several reports suggest that antioxidant enzymes such as SOD1, HO1, GPX1, and G6PD can be activated through increased ROS levels and the increased expression of Nrf2 [51,52,56,57]. Nonetheless, it is clear that OTA induces oxidative stress in the kidney and liver: directly via redox cycling and indirectly via reducing cellular defense involving antioxidants. While these two mechanisms could affect each other, reduced cellular defense may amplify the impact on radicals produced. Consequently, greater susceptibility of the kidney toward oxidative stress can explain the target-specific toxicity of OTA [58,59,60].

### 2.3. Natural Occurrence of OTA

The main organisms involved in the production of OTA are *A. ochraceus*, *A. carbonarius*, and *P. verrucosum*. These ubiquitous fungi are capable of growing and producing OTA at a wide range of temperatures (0–37 °C) and have been found to contaminate agricultural commodities worldwide. Hence, OTA has been found in many raw and processed foods, including cereal grains, beans, coffee, wine, beer, fresh and dried fruits, cocoa, nuts, spices, meats, milk, infant formula, and infant foods, as well as human milk ([7,8,9,10,11,12,13,14,15,16,17,61]. Among all, cereal grains are the largest contributor of OTA in the diet, representing about 55% of the total OTA intake [62]. In an European study, the mean quantity found in cereals was 0.22 ng/g, while the calculated average daily intake was 33 ng/day/person [63]. This was followed by coffee, involved a calculated average daily intake of 6 ng/day/person, with a mean quantity of 0.012 ng/g and an overall contribution of 9%.

In a 3-year national survey of breakfast cereals in the Canadian retail market, OTA was found most commonly in oat-based cereals (63%, 17/27) with the highest concentration of 1.4 ng/g [64]. In a Canadian survey, three out of 25 (12%) oat samples exceeded Health Canada’s proposed regulations for OTA [65]. Kuzdraliński et al. [66] also detected OTA ranging 1.0–5.8 ng/g in 42 out of 71 (59%) samples of oats from Poland. Previously, a two-year national survey had been conducted with 489 breakfast cereal samples collected from the U.S. retail markets and reported 201 (42%) were contaminated with OTA in the range of 0.1–9.3 ng/g [14,15,16,67,68]. The levels of OTA in the most breakfast cereal samples were below the EU limit (3 ng/g) except those of 16 oat-based cereals. The occurrence of OTA was highest in oat-based breakfast cereals (142/203, 70%), followed by wheat-based (38/117, 32%), corn-based (15/103, 15%), and rice-based samples (10/66, 15%). Most significantly, the occurrence and range of OTA found in oat-based infant cereals were 59% and 0.6–22.1 ng/g, respectively; of noting, all of the oat-based infant cereal samples containing OTA exceeded the EU limit for infant cereal (0.5 ng/g), with the highest concentration being 22.1 ng/g.

In the case of OTA occurrence in beers, OTA was detected in 12 out of 19 beers (63%) collected from the United States in the range of 0.01–0.27 ng/mL [61]. De Jesus et al. [69] measured the incidence of OTA in wine collected from the United States and reported that OTA had been detected in 35 out of 41 (85%) of the wines in the range of 0.3–8.6 ng/mL. Soto et al. [70] estimated that the OTA intake from wine was 0.18 ng per day, well below the guidance established by the Scientific Committee on Food (SCF) of 5 ng/kg bw/day, based on a daily consumption of 100 mL/day.

The occurrence of OTA in coffee beans has been described in numerous studies [71,72,73]. According to a survey, OTA was detected in concentrations ranging from 0 to 48 μg/kg in 106 green coffee beans out of 162 samples collected from Africa, America, and Asia [71]. Another study also reported OTA in all the green coffee beans analyzed (n = 47 samples), ranging from 1.3 to 31.5 μg/kg [73]. In addition, the study reported that about 72% of the beans were contaminated with OTA-producing fungi. Martins et al. [72] reported 92% of 60 green coffee beans collected from Brazil were contaminated with OTA-producing fungi and 22 samples (33%) were contaminated with OTA at levels ranging from 0.2 to 7.3 μg/kg. It may also be noted that OTA can frequently be found in ground roasted coffee as well.

### 2.4. Risk Assessment and Regulations 

Due to concerns on the potential risk of OTA having been increasing, especially for infants and young children, authorities in many countries have set measures to monitor/control its level in foods (Table 1). For instance, the EU has set the maximum acceptable limits of OTA for each food group, including cereals and cereal products, roasted coffee, grape juice and wine, dried vine fruit, and foods for infants and young children [74]. Other countries including Brazil, China, India, Korea, and Russia also regulate OTA in foods [75,76,77,78,79]. In Canada, maximum limits for OTA in several commodities and products have been proposed by Health Canada [74]. Future regulation in the U.S. could follow as more information becomes known about the prevalence of this toxin in the country.

Since OTA can contaminate an exceptionally wide variety of agricultural commodities and products, there has been a concern that human exposure may exceed recommended limits [80]. In addition, from processing through storage, OTA is mostly stable in various food products that may be consumed on a daily basis, such as cereal grains, beer, dried fruit (especially raisins), and spices. Although cereals are the main source of OTA (45–50% daily intake), wine is considered the second largest contributor (10–20%) partly due to its consumption frequency [81]. For this reason and due to the relatively long half-life of OTA in human serum, government agencies are increasingly developing regulations to minimize chronic public exposure to the toxin [63]. A more recent risk assessment of OTA based on the two-year nationwide survey data concluded that, in the U.S., OTA exposure is highest among infants and young children consuming oat-based cereals [17].

The maximum tolerable daily intake is very low and is estimated to be between 5.0 and 14.8 ng/kg body weight/day [81]. However, various tolerable intake levels have been estimated through different approaches. For example, the Joint FAO/WHO Expert Committee on Food Additives (JECFA) estimated the level at 100 ng/kg bw/day while the European Commission and Canada provided estimates of 5 ng/kg bw/day and 1.5–5.7 ng/kg bw/day, respectively [82]. The European Food Safety Authority (EFSA) has set a provisional tolerable weekly intake (PTWI) for OTA of 120 ng/kg of body weight [83]. 

EFSA [84] reported the estimated chronic dietary exposure in mean and 95th-percentile levels ranging 0.6–17.8 and 2.4–51.7 ng/kg bw/day, respectively. Based on the survey, they concluded that the most significant contributors were preserved meat, cheese, and grains and their processed products. Moreover, dried/fresh fruit and fruit juices/nectars were also contributing to the exposure in infants and young children, although to a lesser extent than the three major categories. Consequently, different measures have been implemented or proposed for risk management with varying maximum contamination levels for different commodities in different jurisdictions.

## 3. Effects of Food Processing

### 3.1. Non-Thermal Food Processing

Mechanical processes such as sorting and trimming do not destroy OTA but OTA concentrations in foods may be reduced by separation of the part(s) with higher toxin accumulation. There is no operation that destroys mycotoxins in the milling process, but the contamination of mycotoxins including OTA may be redistributed and concentrated in certain milled fractions, such as bran and shorts [85,86]. OTA was found in the highest amounts in germ and bran fractions obtained during the dry milling of wheat, barley, and other cereals [85,86]. Fungal infection generally can lead to damage of the endosperm of grains and results in a higher concentration of mycotoxin being found in smaller particles such as shorts.

As mycotoxins may be bound to cell wall matrix, such as cellulose and protein, mycotoxins may be released by enzymatic hydrolysis through fermentation [87,88]. Therefore, fermentation may cause increased mycotoxin levels in final food products [89,90]. Kupski et al. [91] demonstrated the effect of *Rhizopus oryzae* and *Trichoderma reesei*, which are both non-toxigenic microorganisms, on OTA and observed the degradation of OTA by both fungi in the exponential phase of growth (63.5% reduction after 38 h by *R. oryzae* and 57.7% reduction after 72 h by *T. reesei*, respectively). The research team reported that OTA degradation had a significant correlation with OTα production and carboxypeptidase A (CPA) activity [91].

#### 3.1.1. Physical Separation and Cleaning

The physical separation and cleaning of agricultural commodities after harvest is important as they remove unwanted materials, such as dust, chaff, and small fragments, from agricultural commodities. Manda et al. [92] tried to determine the effect of shelling in cocoa to reduce OTA levels, since the toxin is mainly concentrated in the shells of cocoa beans (92.8%) in comparison with the nib (7.2%). Thus, removing the shell resulted in a significant reduction in OTA, i.e., 49–100%. Similarly, Amézqueta et al. [93] reported that, during the shelling of 22 cocoa bean samples, more than 95% of OTA was removed from 14 samples (64%), while less than a 50% reduction was observed in only one sample.

Scudamore et al. [86] investigated the effect of cleaning using batches (high level 42.2 ng/g and low level 6.1 ng/g) of whole wheat contaminated with OTA by inoculation with *P. verrucosum*. The OTA contamination levels in cleaned wheat fell by about 2–3% in both batches, while the concentrations of OTA measured in subsequent wheat were 53 ng/g and 197 ng/g, respectively, and a quantity representing about 200–300 g was recovered from 130 kg whole wheat [86]. A similar OTA reduction of about 2–3%, by cleaning, was observed in barley [94]. In contrast, scouring, which removes the outer parts of the kernel, was much more effective in reducing OTA from wheat contaminated at low levels (i.e., 27–44% reduction from 6.1 ng/g) than those contaminated at higher levels (i.e., 2.6–8.5% from 42.2 ng/g), corroborating the fact that the accumulation of OTA starts from the surface or outer layer [86]. 

Park et al. [95] determined the effect of washing on OTA reduction in naturally contaminated polished rice and found that about 11.0% of OTA was reduced by water washing (two times of water to rice). Meanwhile, 25.6%, 39.0%, and 42.7% of OTA in rice was removed at one-time washing, two-time washing, and three-time washing, respectively. Mansouri-Nasrabadi et al. [96] also conducted a similar study and reported that about 26% of the OTA in rice was reduced by one-time washing and 39% of OTA was washed by two-time washing, and 43% of OTA decreased by three-time washing. Blanc et al. [97] investigated the effect of cleaning on the reduction in OTA in coffee beans. The coffee bean cleaning process, consists of the elimination of foreign stuffs such as the stones, plant fragments and silverskin of coffee beans by density segregation and air suction, and caused OTA to be reduced by 6.8% from 7.3 ng/g to 6.8 ng/g [97].

A study by Jalili et al. [98] demonstrated the effects of 18 different chemical treatments, including baking soda, acetic acid, and citric acid, which can be used in food systems for OTA reduction in black and white pepper during washing. The peppers were soaked for 2 hr in 2% (*v*/*v*) solutions and then washed using water until the pH was 6.0–6.5. The OTA levels in black and white pepper were reduced by about 20.2% and 16.3%, respectively, when washed with only water, while a significant degree of OTA reduction in black and white pepper washed using acetic acid, citric acid, and baking soda was observed [98]. The OTA loss in black pepper washed using acetic acid, citric acid, and baking soda were 25.8%, 26.2%, and 34.5%, respectively, whereas reductions in OTA in white pepper washed by acetic acid, citric acid, and baking soda were 26.7%, 27.7%, and 38.3%, respectively [98]. Decreased OTA levels in whole beans and bean flour by washing for 2 min were observed around 7% and 39%, respectively [99].

Amézqueta et al. [100] tried to remove OTA from cocoa shells using the solvent extractor ASE 200 with chemicals that can be used for food processing for safety, such as baking soda and other salts rather than organic solvents. When the effects aqueous solutions of 2% sodium bicarbonate or 2% potassium carbonate were compared on the reduction in OTA in cocoa shells, a higher OTA reduction was observed with potassium carbonate (83%) than sodium bicarbonate (27%) [100]. By adjustment of the conditions for the solvent extractor ASE 200 with temperature, pressure and time, about 95% of the OTA naturally present in the cocoa shells was removed [100].

#### 3.1.2. Milling

The effect of milling on OTA reduction using *P. verrucosum*-inoculated wheat batches (high level 42.2 ng/g and low level 6.1 ng/g) was determined by Scudamore et al. [86], and about a 25–33% reduction in OTA in wheat flour was determined. Osborne et al. [101] investigated the effect of milling on the reduction in OTA using two kinds of wheats (soft and hard) inoculated with *P. verrucosum.* It reached a level of 60 ng OTA/g, and two-thirds of OTA loss in the hard wheat and one-third of OTA loss in soft wheat were observed by milling. Peng et al. [102] measured the fate of OTA during milling using wheat grains inoculated with *A. ochraceus,* which, at levels of 93.2 ng/g and 248.3 ng/g, led to a concentration reduction of 43.3–55.2% for OTA, after milling.

#### 3.1.3. Fermentation/Brewing

In brewing beer, OTA may be migrated from contaminated grains, i.e., malted barley or adjuncts, into beer during the brewing process [103]. Barley, which is used in beer production, tends to become contaminated with OTA through OTA-producers’ growth and/or OTA production during the storage or malting process [67]. Similar to other mycotoxins, OTA is relatively stable during fermentation, both alcoholic and malolactic, or brewing, and the reduction in OTA may range from 2 to 100% in the malting and cooking of mash, fermentation and boiling of wort, and final fermentation [103,104,105,106]. Chu et al. [105] investigated the stability of OTA during a conventional micro-brewing process by adding OTA to the raw materials at 1 and 10 μg/g, and found about 72–86% of OTA was reduced during the brewing process. Krogh et al. [104] also demonstrated the fate of OTA during malting and brewing processes by the use of naturally contaminated barley as well as by adding the OTA to the raw materials. In that study, it was reported that moderately contaminated barley (420 and 830 μg of OTA/kg) was used for the malting (germination) process and they found no OTA detected in the malt, while about 2–7% of the initial amount of OTA, resulting in a concentration of 11 to 20 μg/L, was detected in the final beer when using heavily contaminated barley, even though the brewing could be carried out only with the addition of bacterial enzymes because of a very low germination [104]. Nip et al. [106] reported that the contamination level of OTA was reduced by about 14–19% by beer brewing. Scott et al. [103] brewed beers using 0.19 ng/mL of added OTA to the wort and fermentation by three strains of Saccharomyces cerevisiae for 8 days, and found that OTA was decreased by fermentation by 2% to 13%, with straight line slopes over the period. Inoue et al. [107] investigated the fate of OTA during beer brewing and found about 85% of OTA was reduced during processing and about 16% of OTA was detected in yeast crop.

While OTA has also been found in musts and grape juices, information on the effects of unit processes used in winemaking on the fate of OTA in wine is not sufficient. OTA is not likely to be produced during the fermentation since alcohol can effectively inhibit fungal growth [108], but the toxin may be carried over from the contaminated grape to remain throughout fermentation process [109]. Freire et al. [110] showed a reduction of 90.72, 92.44 and 88.15% in the OTA levels for white, rose and red wine, respectively. A study by Csutorás et al. [111] showed OTA reduction in the range of 73–90% in different type of wines (red, rose, and white) during 90-day fermentation in macro-scale experiments using different levels of spiked OTA (0.01–4 µg/mL). The greatest reduction in OTA in red wine was archived (90%), followed by rose wine (86%) and white wine (76%) [111]. Cecchini et al. [112] examined the fate of OTA during the fermentation of white and red musts by using alcoholic fermentation yeast strains and OTA-added musts (2 μg/L). This study showed a significant reduction in OTA in white wines (47–52%) and red wines (53–70%), even though the degree of reduction was different by yeast strains [112]. However, OTA degradation products were not found in this study and the authors concluded that OTA reduction during wine fermentation using yeast strains might have been due to binding or adsorption to the cells. Similarly, Meca et al. [113] also demonstrated the reduction effects of 16 yeast strains of Saccharomyces cerevisiae during wine fermentation and concluded OTA was adsorbed on the external and internal part of the yeast cells.

Yu et al. [114] determined the effect of enzymatic processing on the fate of OTA in grape pomace or OTA in buffer solution. When OTA buffer solution (pH 5.4) was treated with flavozyme, lipase, and carboxypeptidase A at 37 °C for 24 h, 36, 60, and 100% of OTA reduction was observed, respectively, while these enzymes reduced 10.2% (lipase) and 18.3% (carboxypeptidase A) of OTA in grape pomace, and flavozyme did not reduce any OTA [114]. They also showed that this difference was due to the polyphenols in grape pomace that can inhibit different hydrolytic enzymes such as proteaseand lipase [114].

OTA levels in fermented dough were shown to be reduced by about 5.6–12.1% compared with wheat flour [102], whereas Valle-Algarra et al. [87] showed a significant reduction of 29.8–33.5% in OTA during dough fermentation. These different results in OTA reduction by dough fermentation may be related to the method of OTA contamination, as Peng et al. [102] used *A. ochraceus* inoculated wheat while Valle-Algarra et al. [87] used artificially OTA standard spiked wheat. Milani and Heidari [115] fermented wheat flour dough using sourdough (*Lactobacillus casei*, *L. rhamnosus*, *L. acidophilus*, and *L. fermentum*) and three types of yeast based on Saccharomyces cerevisiae (active dry yeast, instant dry yeast, and compressed yeast), and reported that a reduction in OTA during fermentation varied by the type of fermentation methods in the range of 12.9–27.8%, with the highest OTA reduction in compressed yeast.

### 3.2. Thermal Food Processing

Mycotoxins are relatively stable compounds during conventional thermal food processing (80–121 °C) such as boiling, frying, and baking, but certain thermal food processing procedures with high temperatures and pressures, e.g., extrusion processing, can effectively reduce mycotoxin levels and their toxicities. In general, there are lots of important factors related to the degradation of and reduction in mycotoxins in food during food processing, such as the type of food matrix, the initial mycotoxin concentration, the heating temperature together with the time employed, the type of heat transfer, the degree of heat penetration, the pH, the moisture content, the nutritional composition such as fiber and fat levels, the type of additives, etc. [116]. For example, Boudra et al. [117] investigated the degradation of OTA in wheat under different temperatures (100, 150, 200, and 250 °C) and moisture conditions for varying times, and found that the half-lives of OTA were 707, 201, 12, and 6 min at 100, 150, 200, and 250 °C, respectively, for dry wheat and 145, 60, and 19 min, respectively, at 100, 150, and 200 °C for wheat heated under wet conditions. However, the complete elimination of OTA was not observed [117]. Dahal et al. [27] also agreed on OTA’s heat stability and reported that the decomposition of OTA was fastest at pH 10 and slowest at pH 4. However, the effects of thermal food processing on the reduction in OTA in food is not well studied so far in comparison with other mycotoxins such as aflatoxins and fumonisins.

#### 3.2.1. Roasting

Roasting, in general, employs dry heat, often above 160 °C, that may result in a substantial reduction in OTA regardless of the matrix. Hence, the thermal stability of OTA during roasting has been of particular interest to the coffee industry for its prevalence and high levels of contamination in coffee beans. According to previous reports, roasting green coffee beans at 150 °C for 2.5 min was not sufficient to result in a significant reduction in OTA [118], while up to a 90% reduction was achieved during an industrial roasting process reaching up to 250 °C for about 5 min [97,119]. In a study conducted by Nehad et al. [120], roasting coffee beans infected with OTA-producing fungus (*A. ochraceus*) and OTA (30 mg of OTA/kg of coffee beans) at 180 °C for 10 min resulted in a 31% reduction in OTA by using a conventional coffee roaster. In another study with two different concentrations of OTA, i.e., 5.3 and 57.2 μg/kg, produced by culturing *A. westerdijkiae* on coffee beans, the reduction by roasting varied by 7.4–77.6% in low-level OTA beans (5.3 μg/kg), while the maximum reduction in highly contaminated beans (57.2 μg/kg) reached only up to 15.1% [121].

While the roasting process is one of the most well-studied methods among all thermal processes, the reduction in OTA in coffee beans by roasting above 160 °C can vary significantly, ranging from 0% to 97%, as summarized in Table 2. Nonetheless, it seems that an 80% reduction in OTA during-commercial scale coffee roasting at 220–260 °C is achievable despite its largely heterogeneous nature [97]. Although not common, roasting coffee beans at temperatures above 400 °C resulted in widely varying degrees of reduction, i.e., ranging from 0 to 97% [122]. These data suggest that a significant or near-complete reduction in OTA is possible during roasting but is highly dependent on the processing conditions and the level of contamination.

Coffee is not the only commodity commonly subjected to roasting. Manda et al. [92] investigated the effect of roasting on OTA reduction in cocoa. When cocoa beans, cocoa shells, and cocoa nibs were roasted at 200 °C for 25 min, the decrease in OTA levels in cocoa nibs (about 41%) was higher compared to those in cocoa beans (36%) and cocoa shells (24%). Roasting has also been used for cereal grains to improve characteristics such as texture, crispiness, and volume. Moreover, roasted cereal gains have several beneficial effects such as improving digestibility and reducing anti-nutrient factors through the gelatinization of starch and denaturation of protein, enhancing sensory properties such as color and flavor, and extending shelf-life [135].

Lee et al. [126] reported a reduction in OTA ranging from 1.9 to 17.7% in oat grains artificially contaminated with OTA (100 ng/g) during roasting at 120 °C and 180 °C for 30 min and 60 min. In a subsequent study to understand the role of the food matrix during thermal processing, fiber in particular, Carbon and Lee [127] observed significantly lower reduction in OTA in oat-based samples than in rice, corroborating higher incidence and contamination levels in oat-based products. Similarly, a lower reduction in OTA was observed in brown rice (37%) when compared to white rice (60%) during roasting at 160 °C and 200 °C for up to 30 min, suggesting the outer fibrous layer or bran may provide insulation and inhibit heat transfer. 

While roasting is a simple and effective method to reduce OTA in different commodities, the possible formation of degradation products may still be of concern. The degradation products formed during high-temperature roasting (250 °C) include less-toxic diastereomers produced by the partial isomerization of OTA at the C3 position [32]. In general, the disappearance of OTA does not imply an absence or decrease in its toxicity, since the degradation product(s) may still cause adverse effects upon exposure much like their parent compound [136].

#### 3.2.2. Coffee Brewing

Through coffee brewing, it is also possible to reduce OTA contamination levels even it is not significant. Instant coffee brewing using a pressure vessel with hot water at 180 °C showed about a 3% OTA reduction [97]. La Pera et al. [137] demonstrated the fate of OTA during coffee brewing using infusion for 10 min (Turkish coffee making), an Italian moka pot, and a drip brew and Italian moka pot. The latter led to a significant reduction in OTA concentrations of about 51–75%, follow by drip brew (54–73%) infusion for 10 min (17–25%), since the hot water remains in contact with the coffee for a short time. When determining the influence of the brewing process on OTA reduction using a moka, auto-drip, or espresso, the espresso coffee maker showed about a 50% OTA reduction during brewing, followed by moka brewing (32%) and auto-drip (15%) [124]. Malir et al. [138] also conducted a study to demonstrate the reduction in OTA by different methods of brewing such as false Turkish coffee, Turkish coffee, Lungo, Americano, Espresso, Doppio, and Ristretto, and found different degrees of reduction from 34% to 78% depending on the brewing method. Actually, the reduction in OTA in the coffee drink is not because of OTA reduction during the process of coffee brewing, and it might be just a transferal of the OTA content from ground and roasted coffee beans to the coffee drink. It depends on several factors, such as the amount (or volume) of water and ground and roasted coffee beans used and the brewing time (or contact time between the water and ground and roasted coffee beans).

#### 3.2.3. Extrusion

Extrusion, which applies high temperature and pressure with mechanical shear force, is currently used extensively in food industries to produce foodstuffs such as breakfast cereals and infant cereals [139]. Up to now, the effects of this popular and effective food processing method on the destruction of various mycotoxins and the resulting reduction in its toxicity have been well documented [140,141,142], although the reduction effects of extrusion on are not well known so far.

The effects of extrusion processing and conditions on reductions in OTA are summarized in Table 3. According to Scudamore et al. [143], a reduction in OTA in wheat flour during the extrusion process is dependent on the moisture, temperature, and residence time. With a 30% moisture content, the reduction in OTA reached 12% and 24% at 116–120 °C and 133–136 °C, respectively [143]. In barley meal, a reduction in OTA during extrusion ranged 17–86% depending on the varying parameters [144]. The kinetics for OTA reduction by extrusion were also determined to be first-order kinetics, which is a reaction that proceeds at a rate that depends linearly on only one reactant concentration, with the fastest reduction in OTA at 140 °C and a 24% moisture content [144]. A more recent study conducted observed an up to 28% reduction in OTA in oat flakes, with the OTA standard spiked (100 ng/g) by twin-screw extrusion at 180 °C, 20% moisture, 250 rpm screw speed, and a 3 mm die with 193 kJ/kg of specific mechanical energy [145]. The other study showed various reductions in OTA between rice flour (78–82%) and oat flakes (40–43%) with the OTA standard artificially spiked at 100 ng/g by twin-screw extrusion [146]. 

#### 3.2.4. Other Thermal Food Processing

Previous studies have shown a greater reduction in OTA during the baking of biscuits (~65%) in comparison with the baking of bread (0–40%) [86,129,147]. Vidal et al. [148] discovered that the OTA concentration loss was significantly affected by both the baking temperature and time. While significant differences in OTA reduction were not observed between the two initial concentrations (2 and 3 ng/g of flour), a higher reduction in OTA (64%) was observed at 200 °C for 40 min than 21% at 140 °C for 40 min [148]. OTA in wheat flour was reduced during food processing, and they found 90%, 85%, 80%, and 65% of OTA reduced in cake (baked at 220 °C for 20 min), biscuit (baked at 220 °C for 20 min), bread (baked at 220 °C for 35 min), and pasta (boiling for 15 min), respectively [149]. However, these OTA losses were not only from thermal food processing, but also *S. cerevisiae*-yeast-fermented dough was used for the bread, and in the biscuits and cake added baking powder containing baking soda was used [149]. A study on the effect of OTA reduction during baking at 200 °C for 20 min was investigated by Milani and Heidari [115] using several different types of doughs fermented with sourdough culture, instant dry yeast, active dry yeast, and compressed yeast, and a reduction in OTA through baking was observed in the range of 5.3–55.6%, with the highest OTA reduction in compressed yeast. Most recent, a study investigated the stability of OTA during the production of rye bread and wheat pizza bases [150] using a rye flour naturally contaminated with OTA (concentration 6.41 ± 0.52 μg/kg) and yeast. Bryla et al. [150] observed that OTA concentrations in the crust decreased in comparison with the fermented dough by 25.6 and 23% for the bread baked at 180 and 240 °C, respectively, and 8.0–25.4% for the pizza bases, depending on the OTA concentration in the dough. The research team also observed the partial degradation of OTA in the crust of the baked products, which was accompanied by slight OTA racemization: 3.5% of the OTA in the crust of the rye bread baked at 240 °C was transformed into 2*R*′-OTA.

Grape pomace is the residue of grapes after wine making and can be used as an ingredient in food products such as bread and cookies since it contains high amounts of phenolic antioxidants and dietary fiber [151,152,153]. Therefore, Yu et al. (2020) investigated the effect of autoclaving on OTA fate in grape pomace. The autoclaving conditions were at 15 psi and 121 °C for 10, 20, and 30 min, and the reductions in OTA in different varieties of grape pomace were about 19.2–67.6%, 37.6–80.0%, and 43.8–78.0% at 10, 20, and 30 min, respectively [114]. This study also determined the effect of baking at 178 °C for 20 min on OTA fate in a different variety of grape pomace cookies, but what was very interesting was that all cookies showed higher OTA contents in the range of 45.4–69.8% than their corresponding doughs that contained 8.8 ng/g of OTA (dry basis) [114].

Decreased OTA levels in 5 g of whole beans (38 and 48%) and bean flour (27 and 24%) were observed during boiling for 60 min with 25 mL and 40 mL water [99]. In another study, an indirect steaming process, which heated porridges to an 80–85 °C center temperature then maintained them for 10 min for gelatinization, decreased OTA by 59% in rice-based porridges and 14% in oat-based porridges, respectively [134]. OTA in rice can be reduced during ordinary and pressure cooking by 17.3% and 29.0%, respectively [95]. The highest reduction in OTA was observed in cooked rice with excess water (86.6%), followed by normally cooked rice (83.0%) and microwave-oven-cooked rice (82.4%) [154]. Mansouri-Nasrabadi et al. [96] used response surface methodology (RSM) to maximize the degradation of OTA in rice during cooking, measuring the effect of several factors, including the boiling time, salt content, and water-to-rice ratio, on OTA reduction, and then found that the highest OTA loss (76%) was observed at 9.6 min, 3.5% salt, and 4:1 water to rice, respectively.

Some other food processes employing a high temperature and/or pressure showed greater reductions in OTA. The autoclaving of oatmeal (50% water content) resulted in a 74% reduction, while the autoclaving of dry oatmeal or rice cereal (no water added) showed greater losses reaching 87% [131]. Lee et al. [133] determined the effects of direct steam injection (DSI) on the reduction in OTA in oat-based infant cereal; the OTA level in the oat-based infant cereal was reduced by 20% at 85 °C and 28% at 121 °C during the DSI process. DSI is a processing system that exposes a liquid or slurry type of food, such as porridges and infant foods, to a high temperature for short periods of exposure time with high-pressure steam [155]. As the steam (100 °C) had 5.35 times more energy than water (0 °C) and high efficiency in energy transfer through direct contact between the steam and the food matrix [156], the stability of OTA in the oat-based sample was significantly affected by DSI [133]. The OTA levels in rice-based and oat-based porridges during retorting were reduced by 53.8% and 17.2%, respectively [126].

Another report by Lee et al. [133] determined the effects of explosive puffing on OTA in rice and oat grains, i.e., a 15–28% reduction in rice and 38–52% reduction in oats under varying pressures (0.5, 0.7, and 0.9 MPa). The explosive puffing process is a widely used processing system which facilitates the hot-air drying of fruits and vegetables and making snacks [157]. Similar to extrusion processing, explosive puffing processing expands the matrix to a porous structure by the sudden release of pressure generated by superheated steam in a closed chamber [158,159]. Hence, it also could provide an effective measure to reduce OTA levels in foodstuffs and elicit an expected even greater reduction in OTA than extrusion processing, as the explosive puffing process employs a higher temperature and a longer exposure time to high temperature than those of the extrusion process. However, the effect of OTA reduction in oats and rice did not occur as expected. This might be attributed to the high mechanical shear energy, in addition to the high temperature with high pressure, applied in extrusion causing the chemical and molecular transformation of the food components as well as the contaminants.

As OTA in meat products might be reduced by thermal food processing, Pleadin et al. [160] demonstrated the possibility of OTA loss in meat products through the cooking, frying, and baking of several different types of meat sausages including roast, Mediterranean, liver, and blood sausages. About a 7.4% and 12.6% OTA reduction were observed during cooking at 100 °C and frying at 170 °C for 30 min, respectively, while higher OTA reductions were observed during baking for 60 min at 190 °C (70.4%), 200 °C (80.7%), and 220 °C (76.3%) [160]. A study by Josefsson and Möller [161] reported a 20–28% OTA reduction in fried Swedish blood pudding at 150–155 °C for 6–10 min with 5 or 10 mm thickness and a 23–29% OTA loss during frying at 150 °C for 12 min. In the case of kidney, 14–35% OTA reduction was observed when it was boiled at 100 °C for 15 min and then fried at 160 °C for 5 min, while the OTA in adipose tissue, which was fried at 150 °C for 12 min, was not reduced by this heat treatment [161].

### 3.3. Effects of Additives

Based on the literature and technology available to date, it is virtually impossible to achieve complete reduction in or elimination of OTA. Nonetheless, a significant reduction in OTA can be accomplished by optimizing food processing conditions such as using high temperature and pressure, particularly in combination with practical measures to facilitate the mechanism of reduction or degradation. Therefore, it is plausible to explore additional strategies to enhance the degree of OTA reduction and human exposure.

#### 3.3.1. Baking Soda

As Dahal et al. [27] discovered, OTA is very heat-stable under acidic (pH 4) and neutral conditions, while a significant reduction in OTA was observed under alkaline conditions (pH 10) with thermal treatment. Among lots of food additives, baking soda (NaHCO_3_), which has been widely used in baking as a leavening agent, is the only alkaline substance that can be added in food processing. According to the U.S. Food and Drug Administration (FDA), baking soda is considered to be an ingredient generally recognized as safe (GRAS) (21CFR184.1736) [162,163].

Peng et al. [102] determined that the OTA reduction effects in fermented dough through frying were 11.9–16.1% in fermented dough, while the OTA concentration was increased by about 0.7–2.6% by steaming. However, this might not only be as a result of the type of processing (steaming vs. frying) but by adding baking soda before frying. Research was also performed to measure the OTA reduction in cooked noodle, i.e., when boiling wheat flour with baking soda mixed in, which resulted in a gradual decrease in OTA of 12.8–24.0% [102]. In particular, the amount of added baking soda showed a varying effect during indirect steaming on the reduction in OTA in a more recent study by Lee et al. [134] An interesting finding from this study was that the degradation of OTA in rice-based porridge was increased by the added baking soda, even decreasing with 1% of baking soda (59.4% at no additives; 78.1% at 0.5% baking soda, and 68.7% at 1% baking soda), while added baking soda had a greater impact on the oat-based porridge with increasing added baking soda amounts from 13.6% (no additives) to 57.7% and 72.6% with 0.5% and 1% baking soda, respectively [134].

An increased reduction in OTA in a DSI-treated oat-based sample, artificially spiked with OTA standard, by adding baking soda (0.5% and 1% of solid contents) was observed; this was 36.1% and 43.4% at 85 °C and 44.3% and 51.4% at 121 °C, respectively, compared with 19.8% at 85 °C and 27.9 at 121 °C in a sample without baking soda [133]. In the case of the retorting process, the increased degradation of OTA in an oat-based porridge by adding baking soda was greater than that in rice-based porridge [132]. The reduction in OTA in the oat-based porridge was increased from 17.2% (no additives) to 30.3% (0.5% baking soda) and 47.9% (1% baking soda), respectively, while the reduction in OTA in the rice-based porridge was increased from 53.8% (no additives) to 55.5% (0.5% baking soda) and 66.4% (1% baking soda), respectively [132]. Adding baking soda during the extrusion process also induced the increased degradation of OTA [146]. An interesting finding from this study was that OTA in extruded oat-based snacks was decreased with an added amount of baking soda, while such a reduction was not observed in extruded rice-based snacks [146]. The authors analyzed OTA degradation products including the OTA isomer, OTα, and OTα-amide using HPLC-FLD, and reported that the OTA isomer was significantly increased in the extruded rice-based cereals with baking soda, while OTα and OTα-amide were not detected in both types of products.

#### 3.3.2. Sugars

Despite sugars being common ingredients in most foodstuffs, only a little information related to any of their interactions or effects on the fate of OTA during thermal food processing is available so far. For example, a series of investigations were conducted to characterize the reaction between fumonisin B_1_ (FB_1_) and sugars under varying conditions and the toxicity of the reaction products [164,165,166]. An apparent first-order loss of FB1 was observed in a reducing sugar when 6.93 µM FB_1_ was heated in a model system with 100 mM of glucose or fructose in addition to 50 mM of potassium phosphate (pH 7.0) at 80 °C for 48 h [167]. Several researchers discovered significant findings related to the role of reducing sugars, i.e., the formation of FB_1_–sugar complexes via the Maillard reaction and reduced toxicity through blocking of the FB_1_’s amine group by reducing sugars [140,164]. The elucidation of different reaction mechanisms and their significance in food safety is required to provide essential information to set regulatory limits for developing strategies to minimize exposure and toxicity to mycotoxins in foods. While such a Maillard reaction between FB_1_ and reducing sugars is not applicable to OTA, because the amine group for the reaction is not available in OTA, it is critical to understand the role of sugars on the fate of OTA and/or its reaction mechanisms, including characterization of its reaction products, to develop strategies to reduce the risk of exposure and toxicity from OTA.

Gu et al. [168] investigated the thermal stability of OTA in the presence of sugars (glucose, fructose, and sucrose). The addition of fructose (1 mg/mL) resulted in significantly greater losses of OTA in comparison with the process with no added sugar at 150 °C for 50 min or 60 min [168]. An apparent first-order loss of OTA was observed when OTA was heated in the presence of sugars, while no reduction was observed without a reducing sugar [168]. Among those sugars, the half-lives (t_1/2_) and first-order reaction constants indicated that the highest reduction in OTA occurred in the thermal treatment with fructose [168]. Moreover, increased OTα-amide levels, which has no cytotoxicity, and decreased 14-(*R*)-OTA levels, which has a similar cytotoxicity to OTA, were observed by addition of fructose.

In another study, the reduction in OTA was more effective when oat grains (100 ng of OTA/g) were roasted at 180 °C for 30 min with reducing sugars, i.e., glucose (11%) and fructose (15%), compared with the samples roasted with no added sugar (10%) [126]. The reductions in OTA in indirect steamed rice-based and oat-based porridge previously spiked with 20 ng of OTA/g were 59.4% and 13.6% with no additives, respectively, while greater reductions in OTA in rice-based and oat-based porridge were observed with the addition of fructose, and the highest reduction in OTA was observed at 1% fructose addition in both of the commodities [134]. The reduction in OTA spiked (20 μg/kg of dry weight basis) in retorted rice and oat porridge was 54% and 17%, respectively, while a greater reduction in OTA in oat porridge was observed by adding fructose (41% and 36%, respectively); a decreased reduction in OTA in rice porridge was observed with an increased amount of fructose (39% and 18%, respectively) [134]. This suggests that certain sugars may have greater reactivity toward OTA at a given temperature. And it is puzzling to observe such differences, as they may not be explained by their chemical or physical properties. Therefore, there is a need for further research to elucidate their exact reaction mechanisms and characterizations, including the toxicity of the degradation products.

#### 3.3.3. Salts (NaCl)

While salt is one of the most common ingredients in processed foods, only limited data on its effects are available to date. Mansouri-Nasrabadi et al. [96] demonstrated the effect of salt on the reduction in OTA in rice during cooking and found that the OTA level was decreased with an increased salt content, and the greatest reduction in OTA was observed with the addition of 7% (*w*/*w*) of salt.

#### 3.3.4. Organic Acids

Yu et al. [114] examined the effect of organic acids on the reduction in OTA in three grape pomaces of cabernet franc, cabernet sauvignon, and chardonnay, at pH 2 and 37 °C for 24 h. While the results were variable, the reductions in OTA in wine pomace by acetic acid, citric acid, lactic acid, and hydrochloric acid were 59.8, 56.8, −4.4, and 8.1% in cabernet franc, 14.7, 56.8, 67.2, and 62.8% in cabernet sauvignon, and 61.4, 20.2, 46.4, and 25.0% in chardonnay, respectively [114]. Even though the pH was as low as 2 in this study, such low pH levels in foods are rare except lemon juice, beer, vinegar, etc. Hence, more research is necessary to better understand the role of acid in the reduction in OTA, including its reaction mechanism and the possible formation of toxic degradation products.

#### 3.3.5. Adsorbents or Binders

Binders or adsorbents are substances that bind to mycotoxins and prevent absorption during the transition in the gut, and they are mainly studied as feed additives. Consequently, only a limited number of studies are available so far regarding which possible additives can be used during food processing, i.e., for direct human consumption, although they are known to be inert and non-toxic. Mine Kurtbay et al. [169] demonstrated the effects of adsorbents such as bentonite (B), nonylammonium bentonite (NB), dodecylammonium bentonite (DB), KSF-montmorillonite (KSF), and chitosan bead (CB) to reduce the OTA concentration in red wine. The optimum conditions for OTA adsorption from synthetic solutions were at pH 3.5 and at room temperature [169]. In the study, 2.5 ng of OTA/mL of red wine and 250 mg of OTA/mL of synthetic OTA solution were used, and DB and KSF showed the highest adsorption of OTA in the OTA solution (250 mg/mL) while KSF and CB exhibited the highest adsorption of OTA in red wine (2.5 ng/mL) [169]. However, these adsorbents showed a high adsorption capacity for total polyphenols and/or total anthocyanoses. Based on the data, it was concluded that only KSF (250 mg) was effective in adsorbing a high amount of OTA without removing total polyphenols and total anthocyanin from red wine [169].

#### 3.3.6. Combination of Additives

Manda et al. [92] added several additives such as sugar and milk to decrease the OTA content in a cocoa finished product. Roasted and crushed cocoa nibs were ground and mixed with sugar (1:1, *w*/*w*) and then 13.4% of cocoa butter and 10.5% of milk were added to the initial mixture [92]. Since no thermal food processing was involved during the mixing steps with the additives, it did not lead to OTA degradation or removal, but rather a dilution of OTA, i.e., about a 51% decrease in the OTA content in the finished product [92]. 

The combined efficacy of fructose and baking soda during thermal food processing was studied by Lee et al. [132] (Table 4). By adding the two additives in combination with the rice porridge during retorting (0.5% fructose + 0.5% baking soda, *w*/*w*), the reduction level of OTA (36%) did not improve in comparison with reduction by adding individual additives at the same concentration—a 56% OTA reduction by adding 0.5% baking soda and 39% reduction by 0.5% fructose [132]. Similarly, in the retorting of oat porridge, the reduction in OTA by the two additives in combination (40%) was not significantly higher than those by the individual treatments—a 30% reduction by 0.5% baking soda and 41% reduction by 0.5% fructose.

Meanwhile, the OTA reduction when adding combined additives showed different trends during the indirect steaming process. The combination of fructose and baking soda (0.5% + 0.5% *w*/*w*) in rice porridge led to an increased OTA reduction (79%) than the addition of 0.5% baking soda (78%) and 0.5% fructose (63%) alone [134]. In the same study with oat porridge, the combination of fructose and baking soda (0.5% + 0.5% *w*/*w*) led to increased OTA reduction (67%) than 0.5% baking soda (58%) and 0.5% fructose (47%) [134]. Between those two thermal processing technologies of indirect steaming and retorting, the biggest differences were the treatment pressure and treatment time even though the food matrix, solid content and the amount of additives were the same.

### 3.4. Thermal Degradation Products of OTA

While OTA is produced by filamentous fungi, OTA analogues, including thermal degradation products, may be produced by chemical or biological reactions, such as during food processing or metabolism in the body. The most significant concern with OTA analogs is that their degradation products are generally not detected by the conventional methods to detect OTA due to their altered structures. The degradation products of OTA during thermal food processing or metabolism in the body are not well identified or studied. Moreover, purified standards for most of the identified OTA degradation products are not commercially available. Needless to mention, the identification of OTA degradation products and assessment of their toxicities are crucial to calculate more accurate their overall exposure and toxicity levels in our body through food consumption. It should also be noted that a reduced OTA concentration in final food products may not correlate with a reduction in toxicity. Further investigation into the reduction in OTA during thermal food processing is recommended to establish reliable strategies to minimize exposure to OTA, particularly among infants and young children.

Up till now, only a limited number of OTA analogs (Figure 1 and Table 5), including OTα, OTα-amide, and 14-decarbixyl-OTA, have been identified and confirmed for their toxicity [29,30,31,32]. At first, two OTA thermal degradation products, OTA isomer and decarboxy-OTA, were identified by Cramer et al. [32]. Due to high OTA reduction being observed during coffee bean roasting, the authors identified how this OTA was being lost and whether the toxicity of the beans was going up or down [32]. Among 15 roasted coffee samples collected from Germany, the amount of the OTA isomer formed was up to 25.6% relative to OTA, while decarboxy-OTA was produced in traces [32]. The cytotoxicities of these compounds were evaluated using IHKE cells and this showed less toxicity of the OTA isomer (350 nM) and decarboxy-OTA (no cytotoxicity up to 10 µM) compared with OTA (26.2 nM and 16.3 nM, respectively) [32]. The thermal process may also result in the conjugation of OTA with polysaccharides such as OTA-methyl-α-D-glucopyranoside ester, OTA glucose ester, and OTA cellobiose ester [30]. During coffee brewing, however, some of these esters may be hydrolyzed to release OTA, as a higher amount of OTA was detected in brewed coffee [118]. The formation of OTα amide was previously observed when OTA was exposed to light (470 nm) [170], while OTα was formed by the enzyme-catalyzed hydrolysis of the peptide bond in OTA involving lipases and peptidases [26]. Bittner et al. [29] determined the cytotoxicity of OTA reaction products using immortalized human kidney epithelial (IHKE) cell lines and a cell counting kit-8 (CCK-8) assay. While OTα and OTα-amide did not affect the viability of the IHKE cells in the tested concentration range up to 50 µM, OTA (IC_50_ (the half-maximal inhibitory concentration) = 0.5 µM) and 14-(*R*)-OTA (IC_50_ = 4.6 µM) showed cytotoxicity toward IHKE cells. According to Cramer et al. (2010), the non-toxicity of OTα and OTα-amide can be attributed to the missing phenylalanine moiety of OTA, which is responsible for the cytotoxicity of OTA and 14-(*R*)-OTA.

Sueck et al. [171] demonstrated the degradation of OTA and the formation of an OTA isomer during thermal treatment using model heating experiments at 120–260 °C. At 210 °C or above, the formation of the OTA isomer was recorded in less than one minute. While OTA was stable over the entire heating time up to 30 min, with only 3% OTA isomer formed, racemization from OTA to the OTA isomer was observed, with an equilibrium between both compounds being reached in about 20 min [171]. At 30 min at 210, 240, and 260 °C, only 80%, 35%, and 20%, respectively, of the sum of the OTA and OTA isomer were detectable, and screening for decaboxy-OTA and OTα amide revealed them only in trace amounts. The study investigated the incidence of OTA and the OTA isomer in 51 roasted coffee beans collected from France, Germany, and Guatemala, and then reported that OTA was quantified in 96% of the samples in the range of up to 28.4 ng/g, while the OTA isomer was quantifiable in 35% of the samples in the range of up to 3.9 ng/g [171]. Moreover, 30 samples, including 8 of cocoa and 22 of cereal-based products, were also tested, and the OTA isomer was detected in a bread sample and malt coffee powder for the first time.

Since sugars are widely used ingredients in food processing, Gu et al. [168] conducted a study to investigate the fate of OTA during different thermal processes in the presence of sugars, including the formation of degradation products. Sucrose, glucose, and fructose were employed in this study, and fructose was the most effective in reducing OTA when compared to glucose or sucrose. In addition, adding fructose resulted in the highest level of Otα amide, i.e., a non-toxic analog [168]. The most interesting finding in this study was that the OTA isomer was formed at first, then it decreased with an increasing amount of Otα amide [168]. The samples underwent thermal processing with fructose, resulting in a greater amount of OTA degradation products, i.e., mostly Otα amide, when compared to that of the control, in which a more toxic degradation product (OTA isomer) was mainly found [168]. Nonetheless, no OTA isomer, Otα, or Otα amide was detected in roasted oats alone or combined with sugars, glucose and fructose [126]. 

The knowledge about degradation products, especially those formed during food processing, is limited as they are not regulated or detected by routine analysis for mycotoxins in foods such as ELISA, HPLC, and LC-MS. However, considering the need to protect the public from the potent mycotoxin, more work is required to study OTA and its degradation products and update knowledge of their significance in our rapidly evolving society, in which diverse agricultural commodities and food products are increasingly accessible. It also prompts an urgent need to develop analytical methods to detect degradation products and their toxicities along with the availability of authentic standards.

## 4. Conclusions

Among all the strategies and technologies studied to date, it would be logical for the industry to consider thermal processes such as roasting for a higher reduction in OTA, not only as a sole processing method but as a pre-treatment to enhance product quality. In addition, advanced techniques such as extrusion may also provide a practical measure with flexibility and efficiency through the optimization of process variables. Certain food additives may be considered as safe yet practical approaches to facilitate the reduction in OTA in foods, particularly when they are employed in thermal processes. While existing food processing techniques offer varying degrees of reduction, the formation of known and unknown by-products should be considered carefully as they may pose a significant concern to public health.

## Figures and Tables

**Table 1:** Worldwide regulation of ochratoxin A (OTA).

Authority	Commodity	Max. Level of OTA (ng/g)
EU	Unprocessed cereals	5.0
	All product derived from unprocessed cereals intended for direct consumption	3.0
	Dried vine fruits (raisins, currents, sultanas)	10.0
	Roasted coffee beans, ground roasted coffee	5.0
	Instant coffee	10.0
	Beer and wine	2.0
	Grape juice (not concentrated)	2.0
	Infant cereal-based foods, dietary foods for medical purposes	0.5
Codex	Unprocessed cereals of wheat, barley, and rye	5.0
China	Grains and beans	5.0
Singapore	Beer, cereal and cereal products, coffee	3.0
Brazil	Cereals and cereal-based products excl. maize and maize derivates	10.0
	Roasted coffee and soluble coffee	10.0
	Wine	10.0
	Cereal-based baby food	2.0
	Dried fruits	10.0
India	Unprocessed cereals of wheat, barley, rye	20.0
Russia	Wheat, barley, rye, oat and rice cereals and cereal products	5.0
	Specific products for children	0.5
Korea	Grain and their processed foods (grinding, cutting, etc.), coffee beans and roasted coffee	5.0
	Instant coffee	10.0
	Grape juice, concentrated grape juice as reconstituted, wine	2.0
	Raisins	10.0
	Baby foods for infants and young children	0.5
Malaysia	Cereal-based food for infants and children	0.5
	Coffee or ground coffee or coffee powder	5.0
	Instant coffee or soluble coffee, Decaffeinated coffee	10.0
Indonesia	Cereal (rice, corn, sorghum, wheat) and their products	5.0
	Coffee	5.0
	Instant coffee	10.0

**Table 2:** The effects of thermal food processing on ochratoxin A (OTA).

Type of Thermal Processing	Matrix	Reduction (%)	Reference
Roasting	Coffee	0–12%	[123]
	Coffee	17–86%	[119]
	Coffee	16%	[97]
	Coffee	67%	[124]
	Coffee	86–94%	[125]
	Oat	2–18%	[126]
	White/brown rice	15–60%	[127]
	Cocoa beans, shells, and nibs	24–41%	[92]
Explosive puffing	Oat	38–52%	[128]
	Rice	15–28%	
Baking	Wheat	20–40%	[86]
	Wheat	0% (bread), 67% (biscuits)	[129]
Pressure cooking or autoclaving	Bean	Up to 84%	[130]
	Oatmeal, rice	59–89%	[131]
Retorting	Oat	17	[132]
	Rice	54	
Direct steam injection	Oat	20–28%	[133]
Indirect steaming	Oat	13%	[134]
	Rice	59%	
Brewing	Beer (barley)	14–28%	[105]
	Coffee	0–97%	[122]
	Coffee	0–25%	[119]
	Coffee	15–50%	[124]

**Table 3:** The effects of extrusion on ochratoxin A (OTA) reductions in cereal grains.

Type of Extruder	Matrix	Mode of Contamination and Level	MC * (%)	Mass Flow (g/min)	Temp. (°C)	Screw Speed (rpm)	Die Size (mm)	OTA Reduction (%)	Conditions for Maximum OTA Reduction	Reference
Single-screw	Barley meal	*P. verrucosum*; 0.82, 1.43, 0.74 ng/g	24, 27, 30	-	140, 160, 180	40–150	3	17.5–86.5	30% MC, 180 °C, 70 s residence time	[144]
Twin-screw	Wheat flour	*P. verrucosum*; 8.5, 41.1 ng/g	30	600	120, 135	200, 250	4	10.6–39.2	30% MC, 135 °C, 200 rpm	[143]
			17.5		150, 180	350, 400	4	8.3–34.5	17.5% MC, 180 °C, 400 rpm,	
		*P. verrucosum*; 14.2 ng/g	17.6–25	400, 500	160, 180, 200	350, 400	4	21.3–30.7	20% MC, 180 °C, 300 rpm, 400 g/min	
		*P. verrucosum*; 49.8 ng/g	17.6–25	400, 500	180, 200	350, 400	4	31.6–42.2	17.6% MC, 180 °C, 300 rpm, 500 g/min	
Twin-screw	Oat flakes	OTA standard spiking; 100 ng/g	20, 25, 30	66.7	140, 160, 180	150, 200, 250	1.5, 2, 3	0–20	30% MC, 162 °C, 221 rpm, 3 mm	[145]
Twin-screw	Rice flour	OTA standard spiking; 100 ng/g	16	66.7	120, 150	150, 200, 250	3	78.4–82.2	16% MC, 120 °C, 200 rpm	[146]
	Oat flakes		23		160	150, 200, 250	3	39.5–42.7	23% MC, 160 °C, 150 rpm	

* MC: moisture content, wet weight basis.

**Table 4:** The effects of additives during thermal food processing on ochratoxin A (OTA) reduction.

Type of Additives		Type of Processing	Type of Matrix	OTA Contamination Method and Level	Amount of Additives (%) *	OTA Reduction during Thermal Processing			References
						Without Additives	With Additives	Effect of Additives	
Baking soda		Frying	Wheat flour	*A. ochraceus*, 93.2 and 248 ng/g	0.4	46.3 ng/g	38.8 ng/g	↓	[102]
						146.5 ng/g	129.0 ng/g	↓	
		Direct steam injection	Oat flour	OTA standard spiking, 100 ng/g	0.5	19.8–27.9%	36.1–44.3%	↓	[133]
					1		43.4–51.4%	↓↓	
		Twin-screw extrusion	Rice flour	OTA standard spiking, 100 ng/g	0.5	77.9–82.2%	74.9–78.2%	↑	[146]
					1		71.5–77.4%	↑	
			Oat flakes		0.5	39.5–42.7%	56.2–57.7%	↓	
					1		64.6–65.4%	↓	
		Retorting	Rice flour	OTA standard spiking, 20 ng/g	0.5	53.8%	55.5%	=	[132]
					1		66.4%	↓	
			Oat flour		0.5	17.2%	30.3%	↓	
					1		40.8%	↓↓	
		Indirect steaming	Rice flour	OTA standard spiking, 20 ng/g	0.5	59.4%	78.1%	↓	[134]
					1		68.7%	↓	
			Oat flour		0.5	13.6%	57.7%	↓↓↓↓	
					1		72.6%	↓↓↓↓	
Sugar	Glucose	Roasting	Oat grain	OTA standard spiking, 100 ng/g	1	10.0%	11.4%	↓	[126]
	Fructose				1		15.2%	↓	
	Fructose	Retorting	Rice flour	OTA standard spiking, 20 ng/g	0.5	53.8%	38.7%	↑	[132]
					5		18.2%	↑↑↑	
			Oat flour		0.5	17.2%	40.8%	↓↓↓	
					5		35.5%	↓↓	
	Fructose	Indirect steaming	Rice flour	OTA standard spiking, 20 ng/g	0.5	59.4%	62.5%	↓	[134]
					1		80.7%	↓↓	
					5		66.1%	↓	
					10		60.0%	=	
			Oat flour		0.5	13.6%	47.3%	↓↓↓↓	
					1		69.3%	↓↓↓↓	
					5		47.5%	↓↓↓↓	
					10		40.7%	↓↓↓	
Combination	Baking soda + Fructose	Retorting	Rice flour	OTA standard spiking, 20 ng/g	0.5 + 0.5	53.8%	35.8%	↑	[132]
			Oat flour			17.2%	39.8%	↓↓	
	Baking soda + Fructose	Indirect steaming	Rice flour	OTA standard spiking, 20 ng/g	0.5 + 0.5	59.4%	78.6%	↓	[134]
			Oat flour			13.6%	67.2%	↓↓↓↓	
↑	Less than 20% inhibition of reduction								
↑↑	Less than 50% inhibition of reduction								
↑↑↑	More than 50% inhibition of reduction								
=	No reduction								
↓	<1 times reduction								
↓↓	<2 times reduction								
↓↓↓	<3 times reduction								
↓↓↓↓	more than 3-times reduction								

* *w*/*w*, solid basis.

**Table 5:** Thermal degradation products of ochratoxin A (OTA).

Compound	Conditions to Form				Analysis	Ref.
	Initial OTA	Temp (°C)	Time (min)	Additive or Matrix		
14*R*-ochratoxin A	50 μg	175, 200, and 225	5, 10, and 20	Only OTA	HPLC-FLD	[32]
	13 mg	200	10	Only OTA	ESI-MS, LC-MS/MS, NMR	
14-decarboxy-ochratoxin A	50 μg	175, 200, and 225	5, 10, and 20	Only OTA	HPLC-FLD	
14*R*-ochratoxin A	50 μg	225	5	α-D-glucopyranoside	HPLC-FLD	[30]
14-decarboxy-ochratoxin A	50 μg	225	5	α-D-glucopyranoside	HPLC-FLD	
OTA-methyl-α-D-glucopyranoside ester	50 μg	225	5	α-D-glucopyranoside	HPLC-FLD	
	2.5 mg	200	30	α-D-glucopyranoside	ESI-MS, NMR	
	2.0 mg	240	9	Cellulose	ESI-MS	
	5 μg/g of coffee	240	9	Whole coffee beans	LC-MS/MS	
OTA cellobiose ester	2.0 mg	240	9	Cellulose	ESI-MS	
	5 μg/g of coffee	240	9	Whole coffee beans	LC-MS/MS	
14*R*-ochratoxin A	100 μg	200, 220 and 240	9	Only OTA	HPLC-FLD	[29]
14-decarboxy-ochratoxin A	100 μg	200, 220 and 240	9	Only OTA	HPLC-FLD	
ochratoxin α amide	100 μg	200, 220 and 240	9	Only OTA	HPLC-FLD/FTMS	
	214.4 mg	240	20	Only OTA	ESI-MS, NMR, HPLC-ELSD	
ochratoxin α	100 μg	200, 220 and 240	9	Only OTA	HPLC-FLD/FTMS	

## Data Availability

Not applicable.
